# Supplementary material for: Structural and functional characterization of cyclic pyrimidine-regulated anti-phage system
Source: Nat Commun. 2024 Jul 4;15:5634. doi: 10.1038/s41467-024-49861-2 (PMC11224242; doi:10.1038/s41467-024-49861-2)
Supplement: Supplementary file 1 — Supplementary Information [file 41467_2024_49861_MOESM1_ESM.pdf]

Supplementary Tables

Supplementary Table 1. Data collection and refinement statistics of *Ea*PycC crystal*

	***Ea*PycC (PDB 8JSF)**
**Data collection**	
Space group	*I*23
Cell dimensions	
a, *b*, *c* (Å)	164.48, 164.48, 164.48
α, β, γ (°)	90, 90, 90
Resolution (Å)	30–2.20 (2.28–2.20)
Unique reflections	37544 (3697)
Completeness (%)	99.9 (100.0)
Redundancy	33.9 (27.3)
R_{merge} (%)	9.8 (140.4)
R_{pim} (%)	1.7 (27.2)
I/σ *(I)*	51.0 (2.8)
$CC_{1/2}$	0.998 (0.835)
**Refinement**	
No. reflections	37346 (3675)
R_{work} / R_{free}	0.178 / 0.214
No. atoms	
Protein	4078
Water	353
B-factors	
Protein	41.88
Water	44.98
R.m.s. deviations	
Bond lengths (Å)	0.004
Bond angles (°)	0.68

*Numbers in parentheses are for the highest resolution shells.

Supplementary Table 2. Data collection and refinement statistics of *An*PycC crystal*

	***An*PycC (PDB 8JSZ)**
**Data collection**	
Space group	*P*1
Cell dimensions	
a, b, c (Å)	47.66, 50.79, 71.39
α, β, γ (°)	71.21, 83.97, 62.07
Resolution (Å)	30–1.84 (1.91–1.84)
Unique reflections	47377 (4637)
Completeness (%)	97.6 (95.1)
Redundancy	5.5 (5.3)
R_{merge} (%)	6.8 (19.7)
R_{pim} (%)	3.3 (9.2)
I/σ *(I)*	27.5 (11.2)
$CC_{1/2}$	0.998 (0.972)
**Refinement**	
No. reflections	47298 (4189)
R_{work} / R_{free}	0.1695 / 0.2041
No. atoms	
Protein	4168
Ligand/ion	24
Water	664
B-factors	
Protein	21.67
Ligand/ion	34.59
Water	34.13
R.m.s. deviations	
Bond lengths (Å)	0.005
Bond angles (°)	0.75

*Numbers in parentheses are for the highest resolution shells.

Supplementary Table 3. Data collection and refinement statistics of *Np*PycTIR$_{CNBD}$ crystal*

	***Np*PycTIR$_{CNBD}$ (PDB 8JSJ)**
**Data collection**	
Space group	$P6_522$
Cell dimensions	
a, *b*, *c* (Å)	87.12, 87.12, 93.35
α, β, γ (°)	90, 90, 120
Resolution (Å)	30–2.86 (2.96–2.86)
Unique reflections	5229 (502)
Completeness (%)	99.8 (100.0)
Redundancy	34.5 (35.5)
R_{merge} (%)	5.1 (170.4)
R_{pim} (%)	0.9 (28.8)
I/σ *(I)*	85.0 (2.7)
$CC_{1/2}$	0.998 (0.895)
**Refinement**	
No. reflections	4894 (268)
R_{work} / R_{free}	0.268 / 0.332
No. atoms	
Protein	1017
Water	50
B-factors	
Protein	57.46
Water	31.04
R.m.s. deviations	
Bond lengths (Å)	0.016
Bond angles (°)	1.88

*Numbers in parentheses are for the highest resolution shells.

Supplementary Table 4. Data collection and refinement statistics of *Ps*PycTIR$_{CNBD}$–cUMP complex crystal*

	***Ps*PycTIR$_{CNBD}$–cUMP (PDB 8JSK)**
**Data collection**	
Space group	*I*4
Cell dimensions	
a, *b*, *c* (Å)	133.49, 133.49, 34.07
α, β, γ (°)	90, 90, 90
Resolution (Å)	30–2.41 (2.50–2.41)
Unique reflections	12049 (1192)
Completeness (%)	99.8 (100.0)
Redundancy	10.5 (11.0)
R_{merge} (%)	11.4 (98.0)
R_{pim} (%)	3.7 (30.9)
I/σ *(I)*	23.6 (2.7)
$CC_{1/2}$	1.001 (0.808)
**Refinement**	
No. reflections	11953 (1107)
R_{work} / R_{free}	0.208 / 0.219
No. atoms	
Protein	2498
Ligand/ion	20
Water	88
B-factors	
Protein	40.72
Ligand/ion	28.78
Water	35.06
R.m.s. deviations	
Bond lengths (Å)	0.014
Bond angles (°)	1.74

*Numbers in parentheses are for the highest resolution shells.

Supplementary Table 5. The sequences of the codon-optimized genes used in this study.

Elizabethkingia anophelis **PycC$^{\text{wild-type}}$ (*Ea*PycC^{WT})**
ATGGAACAGAAGCTGTATAAGAACTATGCCGATGACATCGCACATTACCTGAAGCAGGGTAAAAAA<br>ATAACCTGCAGAAAGGCCTGAGCTACGAACATTTAGCAAAAATCTGAGCAGCCACCCGAAAATGC<br>AGTGGGTTGATAAAACCAAAAATGAGGCCAATTTCCGCAGCCTGAGCGCCCTGAATACCATTACCGG<br>TCAGATTACCAAATATGAGGAAAAACTGGGCGCACATCCGAGCTTTAGCCATCTGAAAAATACCAAT<br>GATAGCGAGTACCACTATATCGTGAGCATGTTTGTTGACGTGCGTAATAGCACCGGTCTGTTTAAAAA<br>ATTTGACCCGGATGTTGTGGCCAACATTTGTCGTACCATTCAGCTGGCAACCATTCATACCTGTTGGT<br>ATTTTGATGGCTATGTTCACCGTCTGCAGGGTGATGGTCTGATGGTTTATTTGGTGGTAAAGGCACC<br>ACCAAACAGAAAGCAGTTGATAATGCACTGATGGCCGCAAGCTTTATTAGCTATTTTGTGAAAAACG<br>ACCTGAAGAACCTGTTCGAGGAGCAGGGTGTTAGCCGTATTTATACCCGTATTGGTCTGGATTTTGGC<br>GATGATGAAGATACCCTGTGGCATAATGCAGGTATTGGTGAATGTAGCGAAGTTACCACCACCAGCC<br>TGCATACCAGCCTGGCATGTAAAATGCAGGCACAGGCAGAAAGCAATGGTGTTGTTGTTGGTGATAA<br>TATCCTGCCGTATAAGAGCAGCGATAAAAACTATTTACCTACAAGAAGTACAAGAAGAACGGCAGC<br>GAACTGCCGTATGTGTATGAAATTCCGGAAGAATATTTCCGCTACAAGCAGCATGACTTTAACTGGGA<br>GAAATTTCTGAAGAACCACCCGCAGATTCAGGAAGACGAAGATGGTAATCTGACCTTTATTAACCCG<br>AGCCTGCCGCCGAATCCGCGTGTTCAACAAAATATTAATCACCTGCAGCAGAACGTGAGCGGTTATA<br>AACCGTATCTGCGT
Elizabethkingia anophelis **PycC$^{\text{F100A/R142A/Q144A}}$ (*Ea*PycC$^{\text{F100A/R142A/Q144A}}$)**
ATGGAACAGAAGCTGTATAAGAACTATGCCGATGACATCGCACATTACCTGAAGCAGGGTAAAAAA<br>ATAACCTGCAGAAAGGCCTGAGCTACGAACATTTAGCAAAAATCTGAGCAGCCACCCGAAAATGC<br>AGTGGGTTGATAAAACCAAAAATGAGGCCAATTTCCGCAGCCTGAGCGCCCTGAATACCATTACCGG<br>TCAGATTACCAAATATGAGGAAAAACTGGGCGCACATCCGAGCTTTAGCCATCTGAAAAATACCAAT<br>GATAGCGAGTACCACTATATCGTGAGCATGGCAGTTGACGTGCGTAATAGCACCGGTCTGTTTAAAA<br>AATTTGACCCGGATGTTGTGGCCAACATTTGTCGTACCATTCAGCTGGCAACCATTCATACCTGTTGG<br>TATTTTGATGGCTATGTTCACGCCCTGGCAGGTGATGGTCTGATGGTTTATTTGGTGGTAAAGGCAC<br>CACCAAACAGAAAGCAGTTGATAATGCACTGATGGCCGCAAGCTTTATTAGCTATTTTGTGAAAAAC<br>GACCTGAAGAACCTGTTCGAGGAGCAGGGTGTTAGCCGTATTTATACCCGTATTGGTCTGGATTTTGG<br>CGATGATGAAGATACCCTGTGGCATAATGCAGGTATTGGTGAATGTAGCGAAGTTACCACCACCAGC<br>CTGCATACCAGCCTGGCATGTAAAATGCAGGCACAGGCAGAAAGCAATGGTGTTGTTGTTGGTGATA<br>ATATCCTGCCGTATAAGAGCAGCGATAAAAACTATTTACCTACAAGAAGTACAAGAAGAACGGCAG<br>CGAACTGCCGTATGTGTATGAAATTCCGGAAGAATATTTCCGCTACAAGCAGCATGACTTTAACTGGG<br>AGAAATTTCTGAAGAACCACCCGCAGATTCAGGAAGACGAAGATGGTAATCTGACCTTTATTAACCC<br>GAGCCTGCCGCCGAATCCGCGTGTTCAACAAAATATTAATCACCTGCAGCAGAACGTGAGCGGTTAT<br>AAACCGTATCTGCGT
Anabaena sp. **PycC$^{\text{wild-type}}$ (*An*PycC^{WT})**
ATGAAACCGAACCAGTTCCTGAACAGCAGCTTCAGCATCATCAACGAGCAGATCGAAATCTACGAA<br>CGCGGCGTTACCACCCAAAAAGTTAACCAGATCCCGAAAACCGACGATATCCGTATCCAGTCTTCCG

ATAAACCGTGGCATTGGCTGGAAATTCCGGATCTGATCTGCGTCTTCGTCGATATGAAAGGCAGCAC<br>CCAGCTGTCAGTTACCCGTCAAGATCGTACCATGGCATCTGCATACCAGCTGTTTACCAACACCGCG<br>ATTCAGATCTTCCACGATTTCGACACCCCGTACATCGATATCAAAGGCGACGGCGTATTTGCACTGTT<br>CAACAGCAACCAGATCTATCGCGCACTGGCGGCGACCGTTACCTTTAAAACCTTTGTCAAAGAGGTC<br>TTCACCCCGAAAATCAAACAGAAAACCAAAGGCATCATCGTCGGCGGTCATTACGGCATTGACCAG<br>AAAACCGTCCTGGTTCGCAAAATCGGCCTGAAAGTCAACCAGAACCGTCAGGACCCGTATCGTTAC<br>AACGAAGTCTGGGCAGGTAAACCGATTAACATGGCAGCAAAACTGGCGAGCCTGGCAAACATTGAC<br>GAACTGCTGGTTAGCGATCGCTACTTCAACAACCTGAAAAGCGACTTCGTCCTGAAAAGCTGCGGT<br>TGTACCAACGGTATCCCGCAAGAAAACTTCTCCGAACTGTGGCTGCCGAAAAACGTTACCGAAGAA<br>AACAAATTCGACTTCAACAAAGCGTATAGTCTGACCTCTGCTTGGTGTCCGATTCACGGTCGCTTCTA<br>CTGCCAGAACATCCTGAACCTGGACAACGCGAAAAGC
***Anabaena sp.* PycC$^{C221A/C223A/C260A}$ (*An*PycC$^{C221A/C223A/C260A}$)**
ATGAAACCGAACCAGTTCCTGAACAGCAGCTTCAGCATCATCAACGAGCAGATCGAAATCTACGAA<br>CGCGGCGTTACCACCCAAAAAGTTAACCAGATCCCGAAAACCGACGATATCCGTATCCAGTCTTCCG<br>ATAAACCGTGGCATTGGCTGGAAATTCCGGATCTGATCTGCGTCTTCGTCGATATGAAAGGCAGCAC<br>CCAGCTGTCAGTTACCCGTCAAGATCGTACCATGGCATCTGCATACCAGCTGTTTACCAACACCGCG<br>ATTCAGATCTTCCACGATTTCGACACCCCGTACATCGATATCAAAGGCGACGGCGTATTTGCACTGTT<br>CAACAGCAACCAGATCTATCGCGCACTGGCGGCGACCGTTACCTTTAAAACCTTTGTCAAAGAGGTC<br>TTCACCCCGAAAATCAAACAGAAAACCAAAGGCATCATCGTCGGCGGTCATTACGGCATTGACCAG<br>AAAACCGTCCTGGTTCGCAAAATCGGCCTGAAAGTCAACCAGAACCGTCAGGACCCGTATCGTTAC<br>AACGAAGTCTGGGCAGGTAAACCGATTAACATGGCAGCAAAACTGGCGAGCCTGGCAAACATTGAC<br>GAACTGCTGGTTAGCGATCGCTACTTCAACAACCTGAAAAGCGACTTCGTCCTGAAAAGCgcgGGTgc<br>gACCAACGGTATCCCGCAAGAAAACTTCTCCGAACTGTGGCTGCCGAAAAACGTTACCGAAGAAAA<br>CAAATTCGACTTCAACAAAGCGTATAGTCTGACCTCTGCTTGGgcgCCGATTCACGGTCGCTTCTACT<br>GCCAGAACATCCTGAACCTGGACAACGCGAAAAGC
***Anabaena sp.* PycC$^{F60A/K104A/F109A}$ (*An*PycC$^{F60A/K104A/F109A}$)**
ATGAAACCGAACCAGTTCCTGAACAGCAGCTTCAGCATCATCAACGAGCAGATCGAAATCTACGAA<br>CGCGGCGTTACCACCCAAAAAGTTAACCAGATCCCGAAAACCGACGATATCCGTATCCAGTCTTCCG<br>ATAAACCGTGGCATTGGCTGGAAATTCCGGATCTGATCTGCGTCGCGGTCGATATGAAAGGCAGCAC<br>CCAGCTGTCAGTTACCCGTCAAGATCGTACCATGGCATCTGCATACCAGCTGTTTACCAACACCGCG<br>ATTCAGATCTTCCACGATTTCGACACCCCGTACATCGATATCGCGGGCGACGGCGTAGCGGCACTGTT<br>CAACAGCAACCAGATCTATCGCGCACTGGCGGCGACCGTTACCTTTAAAACCTTTGTCAAAGAGGTC<br>TTCACCCCGAAAATCAAACAGAAAACCAAAGGCATCATCGTCGGCGGTCATTACGGCATTGACCAG<br>AAAACCGTCCTGGTTCGCAAAATCGGCCTGAAAGTCAACCAGAACCGTCAGGACCCGTATCGTTAC<br>AACGAAGTCTGGGCAGGTAAACCGATTAACATGGCAGCAAAACTGGCGAGCCTGGCAAACATTGAC<br>GAACTGCTGGTTAGCGATCGCTACTTCAACAACCTGAAAAGCGACTTCGTCCTGAAAAGCTGCGGT<br>TGTACCAACGGTATCCCGCAAGAAAACTTCTCCGAACTGTGGCTGCCGAAAAACGTTACCGAAGAA<br>AACAAATTCGACTTCAACAAAGCGTATAGTCTGACCTCTGCTTGGTGTCCGATTCACGGTCGCTTCTA<br>CTGCCAGAACATCCTGAACCTGGACAACGCGAAAAGC

***Anabaena sp.* PycTM$^{\text{wild-type}}$ (*An*PycTM^{WT})**
ATGCAGAGTAATCAGAGTCAGCTGAGTCCGGAAGAGATCAACGAGAAACTGGTCAACAAACTGCTG<br>ATCATCTTCGCGCTGATCAACGACTGGCTGAAATACGCGGAAGCGAAAAACGCACTGATCATTGCAT<br>TTTGCGGCGCGAGCATTACCGCGATTATTACCTATCTGAGCGCGGTTAACACCATCATCGTCAGCATT<br>GAGCTGAGCATGAAACTGAGCCTGGTCTTCTTCGGCTTCAGCAGCGTTTTTGCGATCATCAGCTTCC<br>TGCCGAAAACCGATAGCGATACCTACTTCTGGAAACTGTTCCACAAATCCAAAAACATGATCCCGGA<br>CACCAGCAAAGATAGCTTCTACTTCTTCGGCCATCTGCGCAAATACGTCGAACTGAAAAACAAACAG<br>CCGAACCCGGATAAACTGCTGGACGCGATTACCCAGTACTATCTGCATCAGAACCCGATCAGCTACA<br>CCAAAGAAGCACGCGACATCTGCATCCAGATCATCAACAACAGCGACATCACCCTGCAGAAATTCAT<br>CCTGAGCGCTTGGTCTGTTCGTTTTGCACTGTGTGCAATTGCGATTACCCCGGTTTGCATGGTTATCA<br>GCCTGATCCTGTACCACCACCTG
***Pseudovibrio sp.* PycC$^{\text{wild-type}}$ (*Ps*PycC^{WT})**
ATGGGTCTGAAAGCGGATCTGGAAAACGAAGTCAAAAAGATTTACAGCAGCACCTGGCAGCGTCGT<br>AACGGTCAAATTGTCCCGGAAGATACCAACCTGCGTTCTGGTAACGACGCTGTTGATCTGGAAGCGA<br>CCATTCTGTACGCGGATCTGGACGAAAGCACCAAACTGGTGGATAACTACAAAGACGAGTTCGCGG<br>CGGAAAACTACAAAACCTTCCTGCAGTGCGCGACCAAAATCATTCGTAGCGAAGGCGGTCATATTCG<br>CTCTTTTGATGGCGATCGCGTTATGGGCATCTACATTGGCAACAGCAAAAATACCAGCGCAGTTCGTA<br>GCGGCCTGAAAATCAACTACGCGGTCAAAAAGATTATCCAGCCGCGTATGAACGCGCAGTACAACA<br>CCAAATACATCATGAAACACGTCACCGGCATCGATTCCACCAAAGTTATGGTCGCGAAAGCGGGTAT<br>TCGCAATAGCAACGATCTGGTTTGGATTGGCAAAGCCGCGAATCATGCAGCAAAACTGTCTGCACTG<br>TCAGCAGATTATCCGACCTGGATCAGCGACAAAGTCTACGACAACATGCACGATAGCGTCAAATACG<br>CGGACGAGAAAAACATGTGGGGTCAACGCGAGTGGGCAGCGCAGAAAAATCGCCGCATTTATCGCT<br>CCACCTATTGGTGGTCCATCGAT
***Pseudovibrio sp.* PycTIR$^{\text{wild-type}}$ (*Ps*PycTIR^{WT})**
ATGATCGAACGCTTTGAAGGCGAAACGGGTATGCGTCTGCTGGTTGAAGCTCTGCAGATGAACAAA<br>ATGGTCCGCGGTAACGTCAAAATCGCGCAGGAACTGGCGAAAAAGATTGTCCTGGAAAAAGTTTCT<br>GCAGGCGACGAACTGATTCAACAGGATACCGAGACCAACGACATCTACTTCATCATCAGCGGTAGCC<br>TGAGCATCATCGTTAACGGCCAGCAGATCGCGATTCGCGGTCCGAACGATCATATTGGCGAAATGGC<br>AGCAATTCAGCCGACCCAAAAACGTTCTGCAACCGTTCAAGCCGTTGAACAGTGTCTGGTTGCGAA<br>AATCACCGAAGCGGATTTCAGCCATCTGGCAAAAAACAACGCGGAGCTGTACAAAAGCATTGCGCA<br>AGAACTGGCACGTCGTCTGCAAGAACGTAACAAACTGGTCACCACCCATCACGAACAGATCCGCGT<br>CTTCATCATCAGTAGCGTTGAAGCGCTGGAAGTTGCACAAGCAATTCAGGCGGCGTTCGAACACGA<br>AAAATTTACCTGTACCGTTTGGACCGACGGCGTTTTTCGTGCAACCAGTTACGCAGTTACCGCACTG<br>GAAGAAGCGATTGACAACAGCGACTTCGCAATTGCAATTGCACACGCAGACGACGTTGCAACCATT<br>CGCGGTCAAGATTGGCCGAGTCCGCGCGATAACGTTATTTTCGAACTGGGCCTGTTTATGGGTCGTCT<br>GGGTCGTAGTCGCGCTATTCTGATGGAACCGCGCGAAGGTAAACTGAAACTGCCGTCTGATATGGCC<br>GGTATCACCACCATTCCGTATCGTTTTGAAGCCGGTAAAGATATGCAGGCGCTGATTGGTCCGGCTTG<br>TCAAAAACTGCGCCTGCACATCAACGAGCTGGGTCCGTACAACGGC
***Pseudovibrio sp.* PycTIR$^{\text{R138A}}$ (*Ps*PycTIR$^{\text{R138A}}$)**

ATGATCGAACGCTTTGAAGGCGAAACGGGTATGCGTCTGCTGGTTGAAGCCCTGCAAATGAACAAA
ATGGTCCGCGGCAACGTCAAAATCGCACAGGAACTGGCGAAAAAGATTGTCCTGGAGAAAGTTAGC
GCAGGCGACGAACTGATTCAGCAGGATACCGAAACCAACGACATCTACTTCATCATCAGCGGTAGCC
TGAGCATCATCGTTAACGGTCAGCAGATTGCAATTCGCGGTCCGAACGATCATATTGGCGAAATGGC
AGCAATTCAGCCGACCCAAAAACGTTCTGCAACCGTTCAAGCCGTTGAACAGTGTCTGGTTGCGAA
AATCACCGAGGCGGATTTCAGCCATCTGGCGAAAAACAACGCGGAGCTGTACAAAAGCATTGCGCA
AGAACTGGCACGTGCACTGCAAGAACGTAACAAACTGGTCACCACCCATCACGAACAGATCCGCGT
TTTCATCATCAGCAGCGTTGAAGCGCTGGAAGTTGCACAAGCGATTCAGGCGGCGTTTGAACACGA
AAAATTCACCTGTACCGTTTGGACCGACGGCGTTTTTCGCGCAACCAGTTACGCAGTTACCGCACTG
GAAGAAGCGATCGATAACAGCGACTTCGCGATTGCAATTGCACACGCAGACGACGTTGCAACCATT
CGCGGTCAAGATTGGCCGAGTCCGCGCGATAACGTCATCTTTGAGCTGGGCCTGTTCATGGGTCGTC
TGGGTCGTAGTCGCGCTATTCTGATGGAACCGCGCGAAGGTAAACTGAAACTGCCGTCTGATATGGC
CGGTATTACCACCATTCCGTACCGCTTTGAAGCCGGCAAAGATATGCAGGCACTGATTGGTCCGGCTT
GTCAAAAACTGCGTCTGCACATCAACGAGCTGGGTCCGTATAACGGC

***Pseudovibrio sp.* PycTIR^{N143A} (*Ps*PycTIR^{N143A})**

ATGATCGAACGCTTTGAAGGCGAAACGGGTATGCGTCTGCTGGTTGAAGCCCTGCAAATGAACAAA
ATGGTCCGCGGCAACGTCAAAATCGCACAGGAACTGGCGAAAAAGATTGTCCTGGAGAAAGTTAGC
GCAGGCGACGAACTGATTCAGCAGGATACCGAAACCAACGACATCTACTTCATCATCAGCGGTAGCC
TGAGCATCATCGTTAACGGTCAGCAGATTGCAATTCGCGGTCCGAACGATCATATTGGCGAAATGGC
AGCAATTCAGCCGACCCAAAAACGTTCTGCAACCGTTCAAGCCGTTGAACAGTGTCTGGTTGCGAA
AATCACCGAGGCGGATTTCAGCCATCTGGCGAAAAACAACGCGGAGCTGTACAAAAGCATTGCGCA
AGAACTGGCACGTCGTCTGCAAGAACGTGCAAAACTGGTCACCACCCATCACGAACAGATCCGCGT
TTTCATCATCAGCAGCGTTGAAGCGCTGGAAGTTGCACAAGCGATTCAGGCGGCGTTTGAACACGA
AAAATTCACCTGTACCGTTTGGACCGACGGCGTTTTTCGCGCAACCAGTTACGCAGTTACCGCACTG
GAAGAAGCGATCGATAACAGCGACTTCGCGATTGCAATTGCACACGCAGACGACGTTGCAACCATT
CGCGGTCAAGATTGGCCGAGTCCGCGCGATAACGTCATCTTTGAGCTGGGCCTGTTCATGGGTCGTC
TGGGTCGTAGTCGCGCTATTCTGATGGAACCGCGCGAAGGTAAACTGAAACTGCCGTCTGATATGGC
CGGTATTACCACCATTCCGTACCGCTTTGAAGCCGGCAAAGATATGCAGGCACTGATTGGTCCGGCTT
GTCAAAAACTGCGTCTGCACATCAACGAGCTGGGTCCGTATAACGGC

***Pseudovibrio sp.* PycTIR^{N143S} (*Ps*PycTIR^{N143S})**

ATGATCGAACGCTTTGAAGGCGAAACGGGTATGCGTCTGCTGGTTGAAGCTCTGCAGATGAACAAA
ATGGTCCGCGGTAACGTCAAAATCGCGCAGGAACTGGCGAAAAAGATTGTCCTGGAAAAAGTTTCT
GCAGGCGACGAACTGATTCAACAGGATACCGAGACCAACGACATCTACTTCATCATCAGCGGTAGCC
TGAGCATCATCGTTAACGGCCAGCAGATCGCGATTCGCGGTCCGAACGATCATATTGGCGAAATGGC
AGCAATTCAGCCGACCCAAAAACGTTCTGCAACCGTTCAAGCCGTTGAACAGTGTCTGGTTGCGAA
AATCACCGAAGCGGATTTCAGCCATCTGGCAAAAAACAACGCGGAGCTGTACAAAAGCATTGCGCA
AGAACTGGCACGTCGTCTGCAAGAACGTTCTAAACTGGTCACCACCCATCACGAACAGATCCGCGT
CTTCATCATCAGTAGCGTTGAAGCGCTGGAAGTTGCACAAGCAATTCAGGCGGCGTTCGAACACGA
AAAATTTACCTGTACCGTTTGGACCGACGGCGTTTTTCGTGCAACCAGTTACGCAGTTACCGCACTG

GAAGAAGCGATTGACAACAGCGACTTCGCAATTGCAATTGCACACGCAGACGACGTTGCAACCATT CGCGGTCAAGATTGGCCGAGTCCGCGCGATAACGTTATTTTCGAACTGGGCCTGTTTATGGGTCGTCT GGGTCGTAGTCGCGCTATTCTGATGGAACCGCGCGAAGGTAAACTGAAACTGCCGTCTGATATGGCC GGTATCACCACCATTCCGTATCGTTTTGAAGCCGGTAAAGATATGCAGGCGCTGATTGGTCCGGCTTG TCAAAAACTGCGCCTGCACATCAACGAGCTGGGTCCGTACAACGGC
***Pseudovibrio sp.* PycTIR^{E114R} (*Ps*PycTIR^{E114R})**
ATGATCGAACGCTTTGAAGGCGAAACGGGTATGCGTCTGCTGGTTGAAGCCCTGCAAATGAACAAA ATGGTCCGCGGCAACGTCAAAATCGCACAGGAACTGGCGAAAAAGATTGTCCTGGAGAAAGTTAGC GCAGGCGACGAACTGATTCAGCAGGATACCGAAACCAACGACATCTACTTCATCATCAGCGGTAGCC TGAGCATCATCGTTAACGGTCAGCAGATTGCAATTCGCGGTCCGAACGATCATATTGGCGAAATGGC AGCAATTCAGCCGACCCAAAAACGTTCTGCAACCGTTCAAGCCGTTGAACAGTGTCTGGTTGCGAA AATCACCCGTGCGGATTTCAGCCATCTGGCGAAAAACAACGCGGAGCTGTACAAAAGCATTGCGCA AGAACTGGCACGTCGTCTGCAAGAACGTAACAAACTGGTCACCACCCATCACGAACAGATCCGCGT TTTCATCATCAGCAGCGTTGAAGCGCTGGAAGTTGCACAAGCGATTCAGGCGGCGTTTGAACACGA AAAATTCACCTGTACCGTTTGGACCGACGGCGTTTTTCGCGCAACCAGTTACGCAGTTACCGCACTG GAAGAAGCGATCGATAACAGCGACTTCGCGATTGCAATTGCACACGCAGACGACGTTGCAACCATT CGCGGTCAAGATTGGCCGAGTCCGCGCGATAACGTCATCTTTGAGCTGGGCCTGTTCATGGGTCGTC TGGGTCGTAGTCGCGCTATTCTGATGGAACCGCGCGAAGGTAAACTGAAACTGCCGTCTGATATGGC CGGTATTACCACCATTCCGTACCGCTTTGAAGCCGGCAAAGATATGCAGGCACTGATTGGTCCGGCTT GTCAAAAACTGCGTCTGCACATCAACGAGCTGGGTCCGTATAACGGC
**CNBD domain of *Novosphingobium pentaromativorans* PycTIR (*Np*PycTIR$_{CNBD}$)**
ATGGGTGCACTGCTGGATCGTTTTAGCGGTGATGAAAAAAAAGGCCGTCTGATTGAAGCAGTTTGCG GCCAGGATCTGGTTGCAAATGATAAAGATCTGGCAGAACAGATTGTGGCCGCAGGTGTTCTGCAGG AAATTGCAGATGGTGATGTTATTATTAAGCAGGGCGACTGGGATGATGACCTGTTTCTGATTCTGGCA GGTAAAATGCAGATTACCATTAACGGCCGTCCGCAGACCGTTCGTGAAGCAGGTACACATGTTGGTG AACTGACCGGTACCAGCCCGGCACGTCCTCGTACAGCAACAGTTAGTGCAATTGGTGAAGCACTGG TTCTGCGTCTGAAACGTACCGTTCTGGATGAAATTACCCGTGATAGCCCGGCATATCTGAAACGTATG CTGGATGTTGTTGCAGGTCGTCTGGATGAACGTAATAAAGGTATTGGT
**CNBD domain of *Acinetobacter haemolyticus* PycTIR (*Ah*PycTIR$_{CNBD}$)**
ATGAAACTGATCGAACGCTTCATCAGCAACGAGACCGCAGTTATTAACGCGCTGCTGAATCAGAAAA GCATCCCGAGCCAGGAAATCGCGAAAGATATCGCGGAGAAAGGCGAAGTTGTTAGCTACCAGAGCG GCGAATACATCATCAAACAGGGCGACTACGATCAGGACGTTTACTACATCCTGGCGGGTAAAGTCGA ACTGCACATTAACGGCGTTGTTCTGCCGTACGAACGCGGCGAAGATGTTAGCGTTGGCGAACTGTCT GCGATTAATGCAAGTCAAGCACGTACCGCAAGTCTGCTGGTTACCTCTGAAACCGTTGCGCTGAAAA TCAAACCGGACGACTTCCAGACCATTCTGCGTAAACATCCGGAGGTCAGCATGTTCCTGCTGAAAG ACGTTAGCTCTCGTCTGGCACAACGTAACGATCTGATCAACAAATGCAAC
**CNBD domain of *Stenotrophomonas maltophilia* PycTIR (*Sm*PycTIR$_{CNBD}$)**
ATGGCAGCAAGCATTGTTGAACGTCTGTCTGCAGATGGCGGTCGTCGTCTGGTAGCAGCACTGGCA GAATTTCGTACCCTGGCTGGTCTGGAAGGTGCAGCAGTTCAACTGGCTGAAGCAGGTGAACTGCTG

GAAGTTCCGGCTGGGGGTAGTTTTATTACCCAAGACGACGCCGAAACCGACGTGTTTTTCATTGTTG
CGGGCGCCGTTAACGTCGTGGTCAACGGCAAAGTTGTTAACACCCGTCGCGTAGGCGATCACGTTG
GCGAAATGGCAGCAATTGAACCGACCCAACTGCGCGCAGCAACCATTACCGCAACCGAACCGACCG
TTGTTCTGAAAGTCCCGGAAAGCGATTTCAGCCGTATTGCAGACGCACATCCGGTTATTTGGCGTCG
TCTGGCAGCAACCCTGAGTCGTCGTCTGAAAGAACGTAACCGCATGATCACC

Supplementary Figures

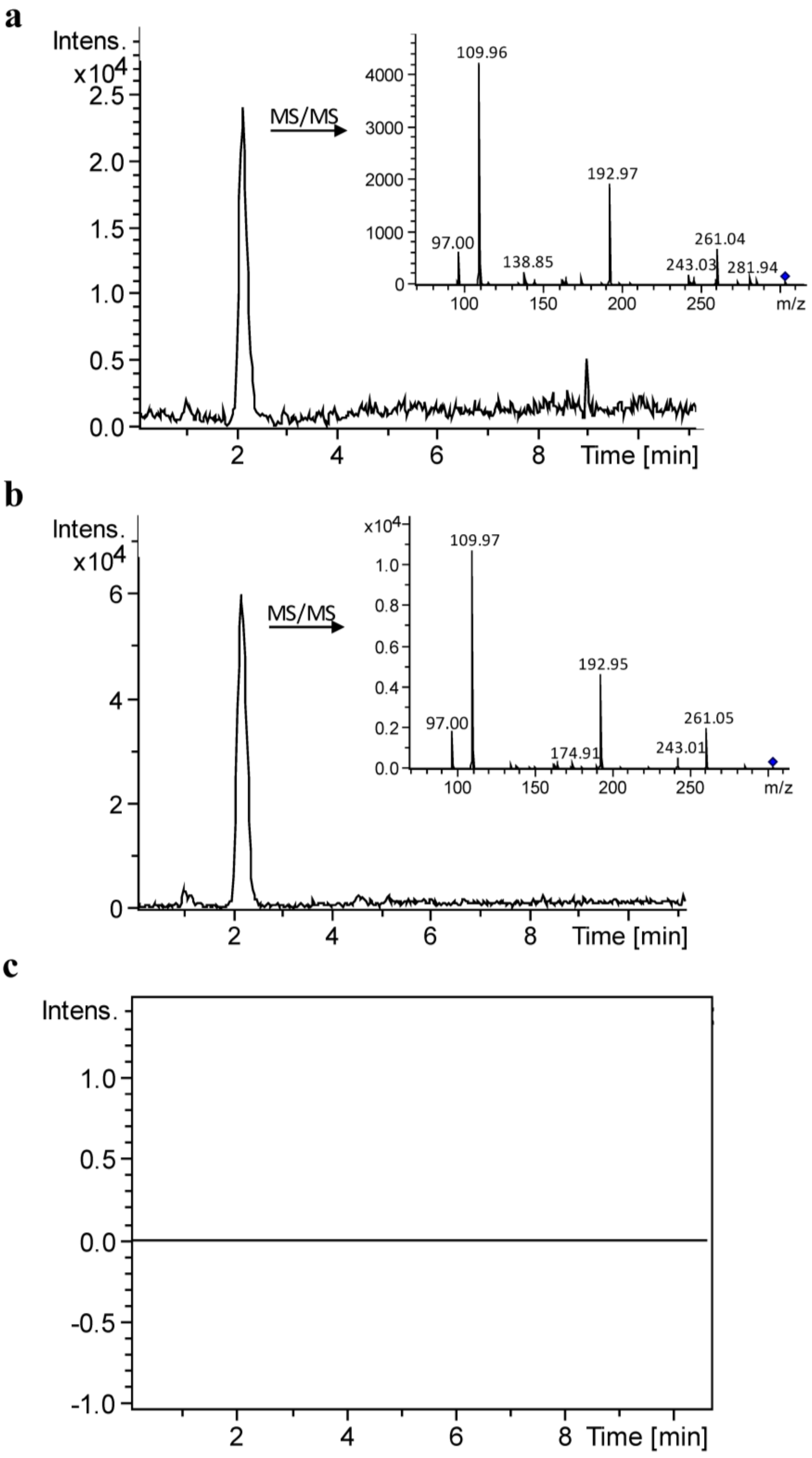

Supplementary Figure 1. Extraction ion chromatography (m/z 304.0→m/z 109.9) and MS/MS spectrum of LC-MS/MS analysis of the cCMP products synthesized by *Ea*PycC.

(a) LC-MS/MS analysis of the chemical standard of cCMP.

(b, c) LC-MS/MS analysis of cCMP produced in the overnight reaction containing 1 mg/ml (b) *Ea*PycC^{WT} or (c) *Ea*PycC$^{F100A/R142A/Q144A}$ with 1 mM CTP, 1 mM Mg^{2+}, and 1 mM Mn^{2+} at pH 9.0. The cCMP signal is not observed in (c).

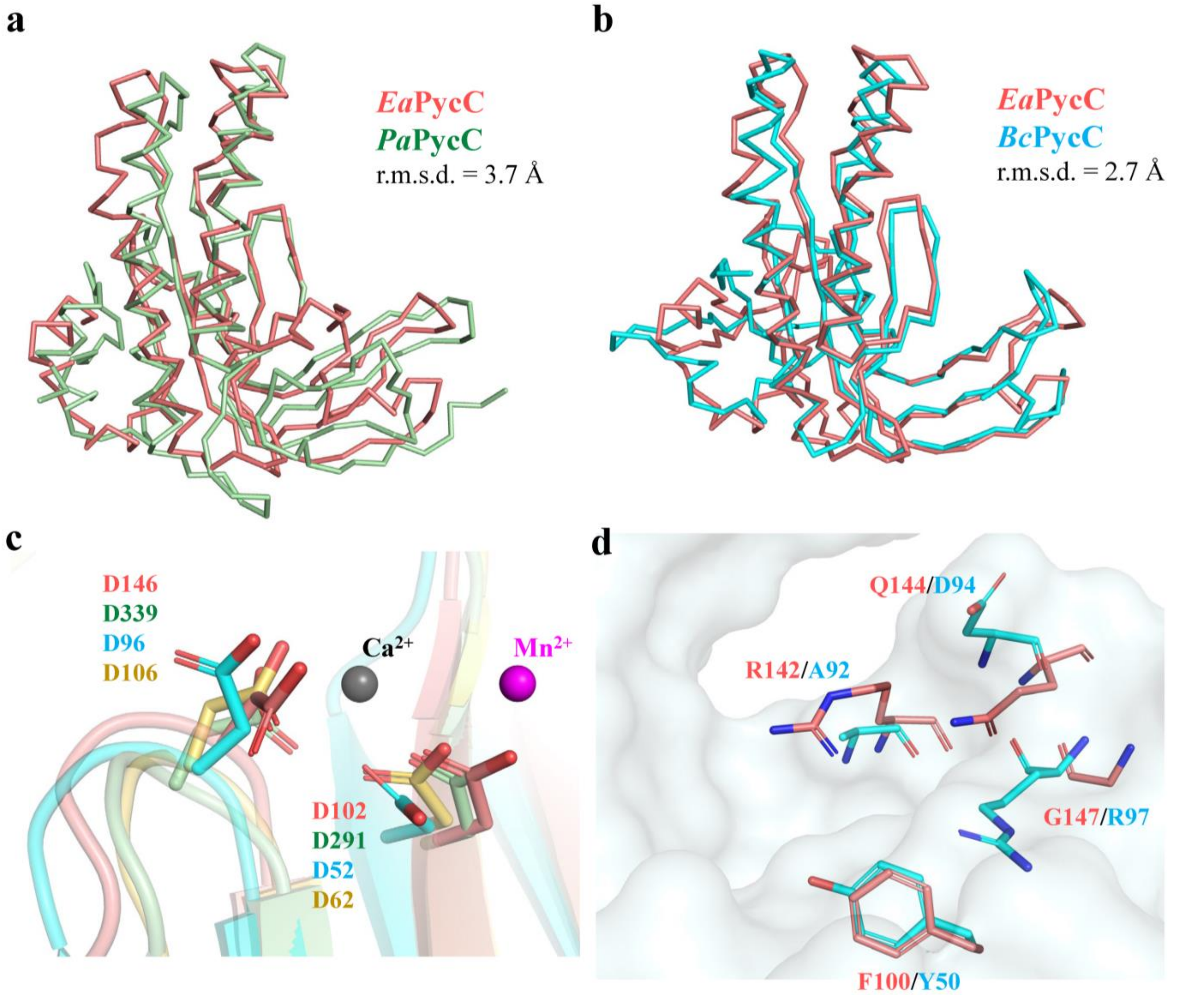

Supplementary Figure 2. Structural comparison of *Ea*PycC (salmon) with *Pa*PycC (pale green, PDB 6YII) and *Bc*PycC (cyan, PDB 7R65).

Ribbon representation of the superimposed *Ea*PycC with (a) *Pa*PycC and (b) *Bc*PycC using DALI server. For clarity, only the nucleotide cyclase core of them is shown. (c) Superimposed catalytic aspartate residues of *Ea*PycC with those in *Pa*PycC, *Bc*PycC and *An*PycC model (yellow-orange). The calcium and manganese ion in the *Pa*PycC structure are shown in grey and magenta sphere, respectively. (d) Superimposed active-site residues of both *Ea*PycC and *Bc*PycC.

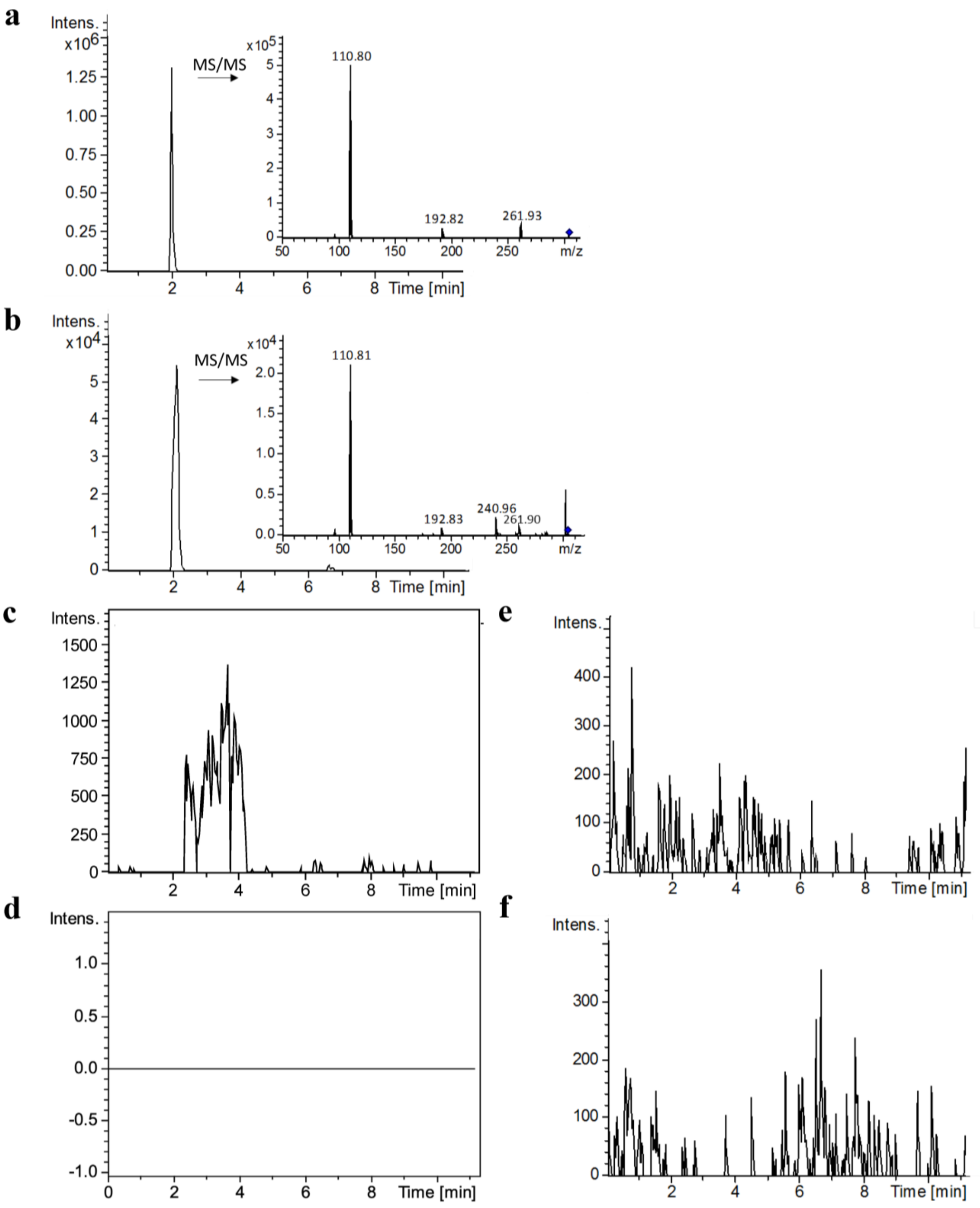

Supplementary Figure 3. Extraction ion chromatography (m/z 305.0→m/z 110.8) and MS/MS spectrum of the LC-MS/MS analysis of the cUMP products synthesized by *An*PycC.

(a) LC-MS/MS analysis of the chemical standard of cUMP. (b–d) LC-MS/MS analysis of cUMP produced in the overnight reaction containing 1 mg/ml *An*PycC^{WT}, 1 mM UTP, 1 mM Mg^{2+}, and 1 mM Mn^{2+} at (b) pH 9.0, (c) pH 8.0, and (d) pH 6.5. (e, f) LC-MS/MS analysis of cUMP produced in the overnight reaction containing 1 mg/ml (e) *An*PycC$^{C221A/C223A/C260A}$ or (f) *An*PycC$^{F60A/K104A/F109A}$ with 1 mM UTP, 1 mM Mg^{2+}, and 1 mM Mn^{2+} at pH 9.0. The cUMP signals are not observed in (c–f).

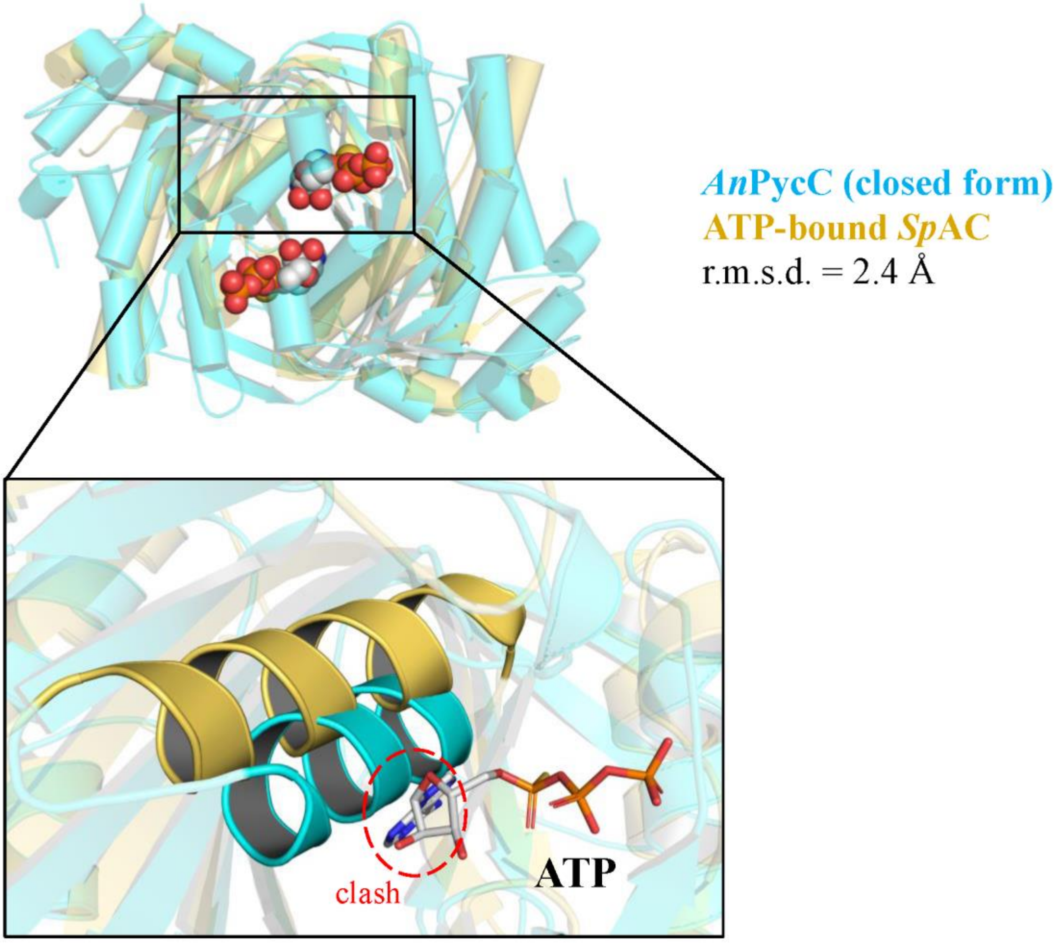

Supplementary Figure 4. Structural comparison of *An*PycC with ATP-bound *Sp*AC.

Top, superimposition of *An*PycC dimer structure (cyan) determined in this study with *Spirulina platensis* adenylyl cyclases (*Sp*AC, PDB: 1CW0, yellow-orange). Bottom, the enlarged view of the superimposed active sites. The bound ATP in *Sp*AC will make serious clashes with α5 helix of *An*PycC.

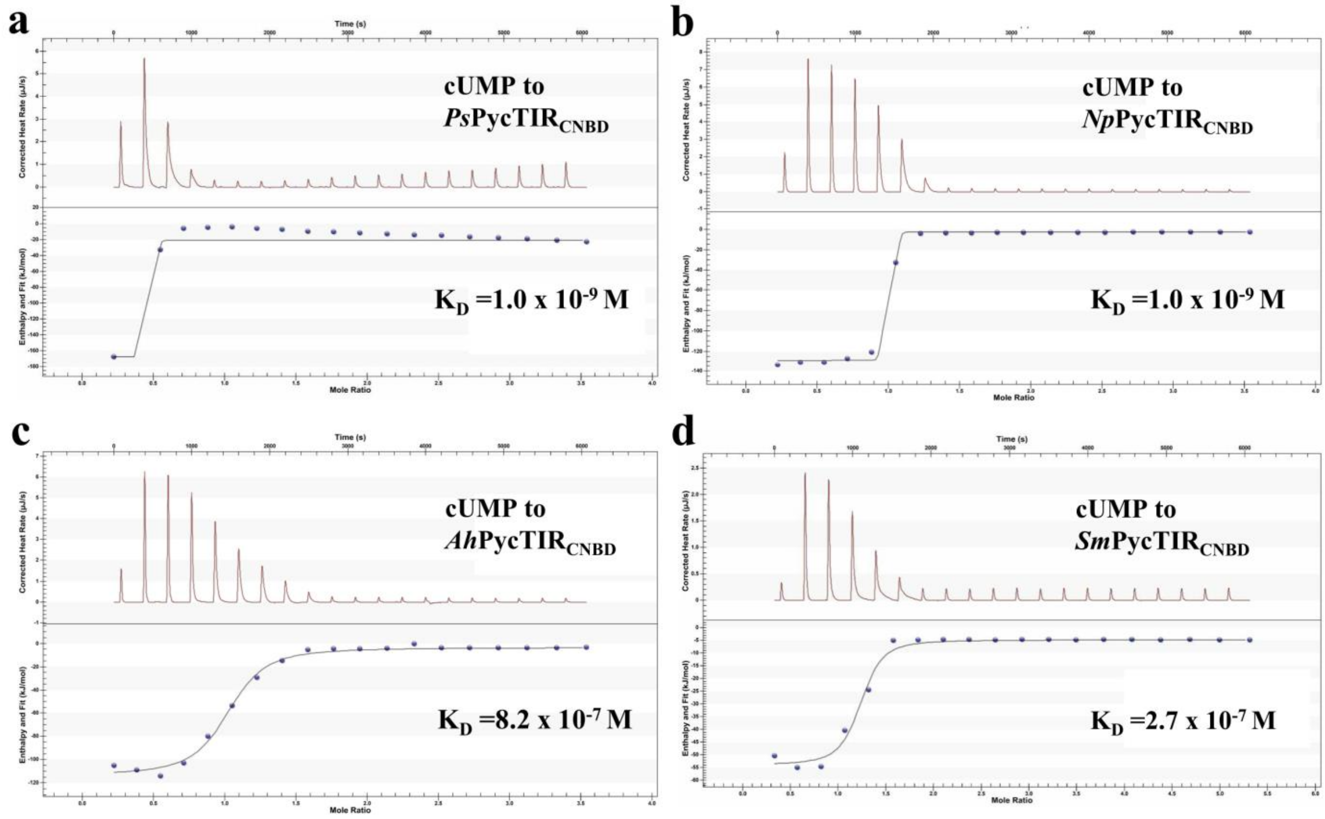

Supplementary Figure 5. ITC analysis of cUMP binding to different PycTIR proteins.

ITC analysis of cUMP binding to (a) *Ps*PycTIR$_{CNBD}$, (b) *Np*PycTIR$_{CNBD}$, (b) *Ah*PycTIR$_{CNBD}$, and (c) *Sm*PycTIR$_{CNBD}$. The dissociation constants between them are indicated. The ITC experiments were repeated twice and the representative of them are shown.

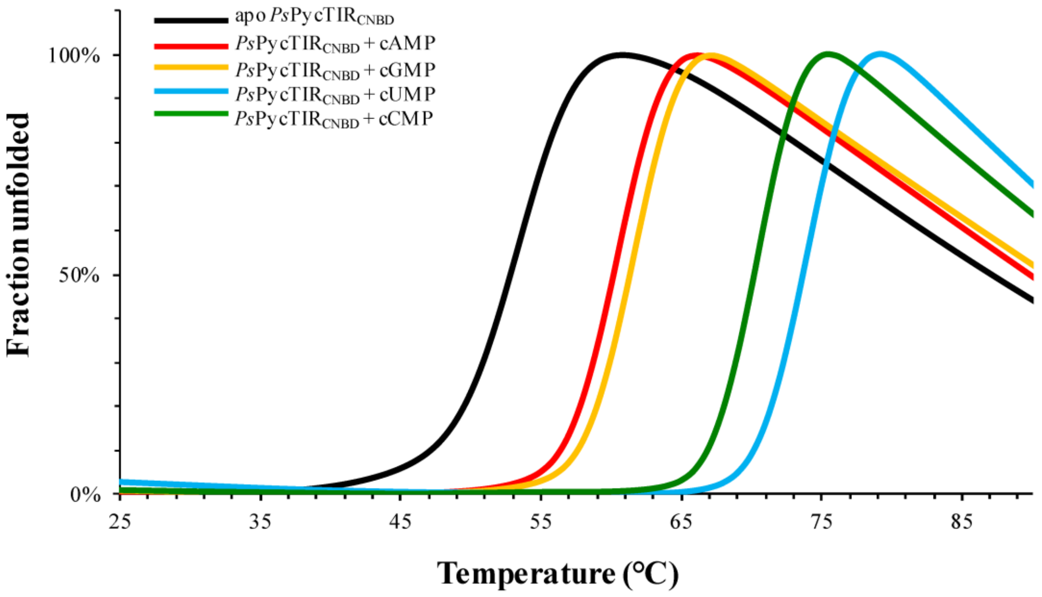

Supplementary Figure 6. Thermal shift assay for cyclic nucleotides binding to the CNBD domain of *Ps*PycTIR proteins.

Thermal shift analysis of *Ps*PycTIR$_{CNBD}$ in the absence (black line) or presence of cAMP (red), cGMP (yellow), cUMP (cyan), and cCMP (green). The experiments were repeated at two times and the representative of them are shown.

a

b

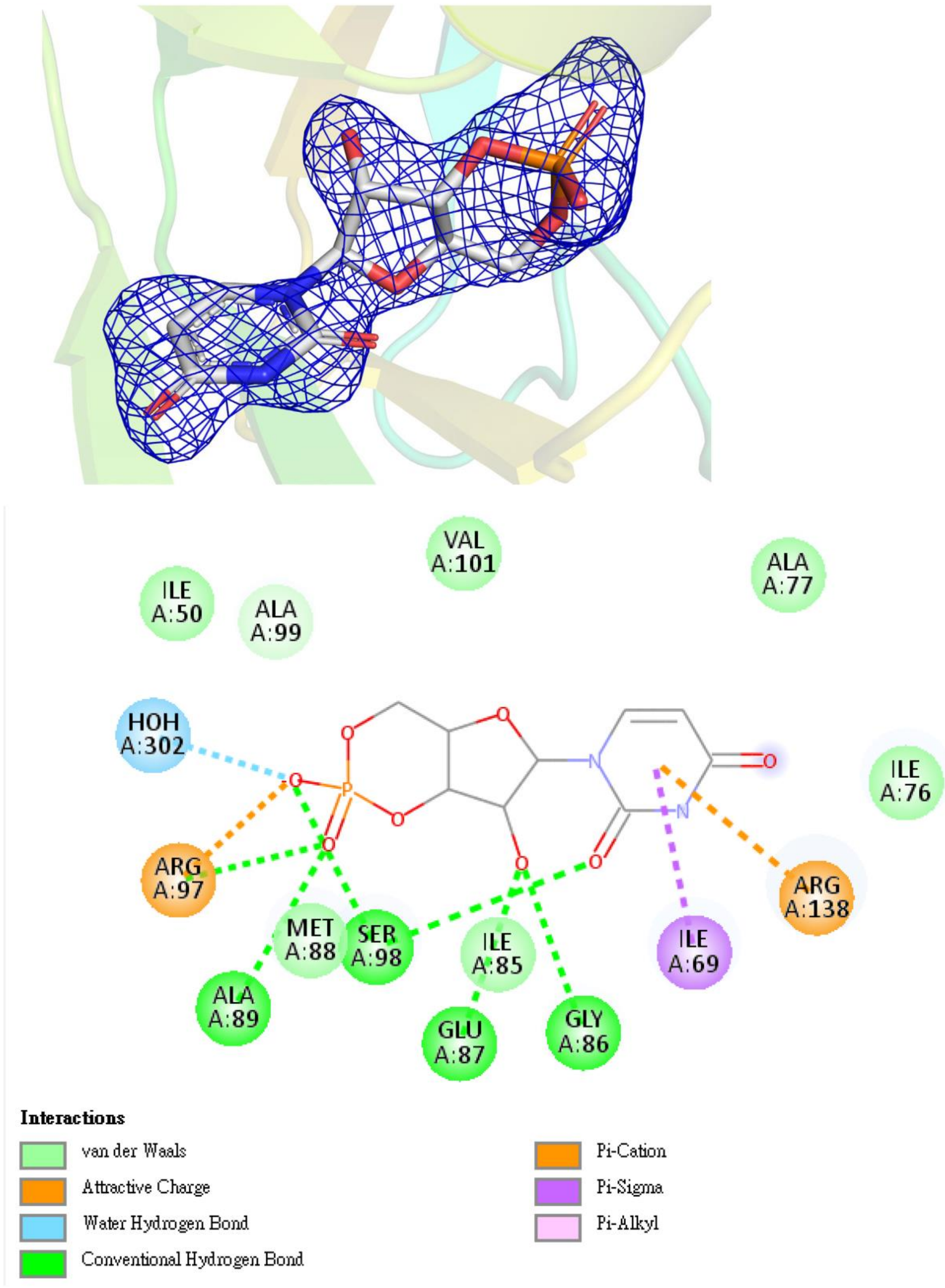

Supplementary Figure 7. Detailed analysis of the bound cUMP in *Ps*PycTIR$_{CNBD}$ complex structure. (a) *F*o-*F*c omit electron-density map of the bound cUMP contoured at 3-σ level and shown in blue mesh. (b) The detailed interactions between the bound cUMP and *Ps*PycTIR$_{CNBD}$. Residues are colored based on the interaction type as indicated.

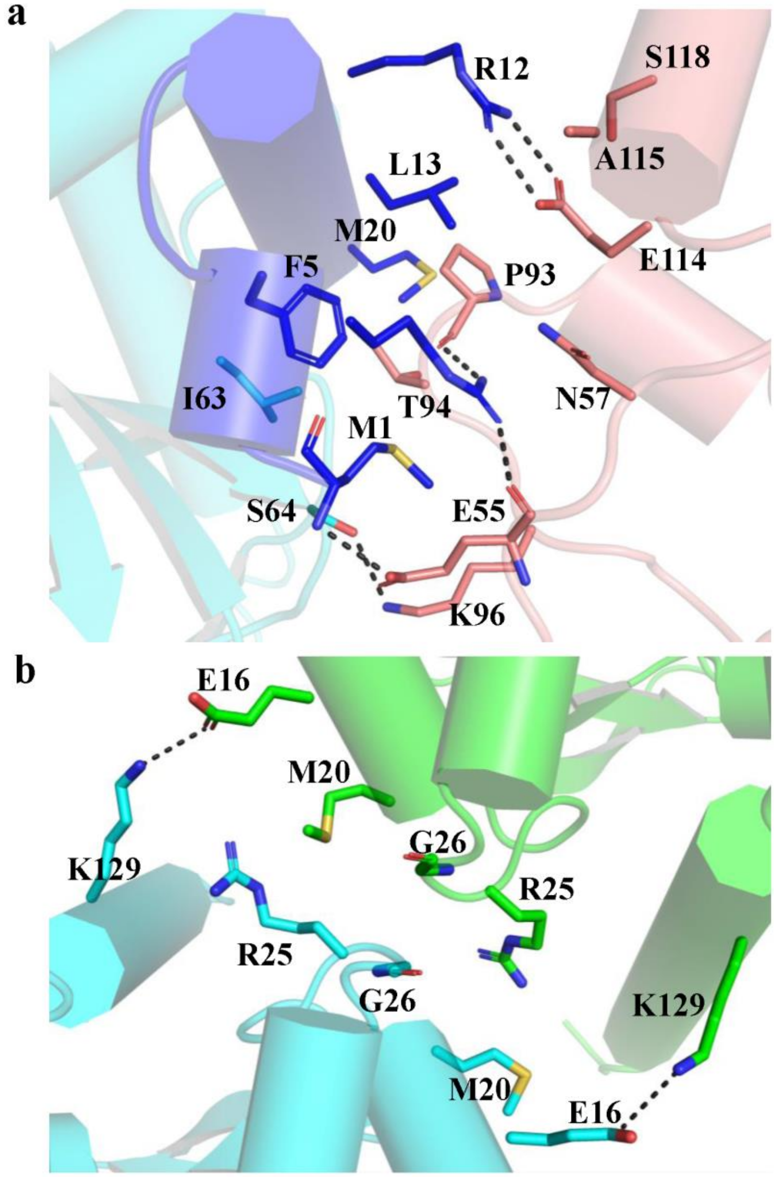

Supplementary Figure 8. Detailed analysis of *Ps*PycTIR oligomerization implicated by crystal packing.

(a) The detailed interactions between protomer A (cyan and blue) and protomer A' (salmon) of *Ps*PycTIR as shown in Figure 6. (b) The detailed interactions between protomer A (cyan) and protomer B' (green) of *Ps*PycTIR as shown in Figure 6. The interacting residues are shown in sticks and indicated. The H-bonds are shown in black dashed lines.

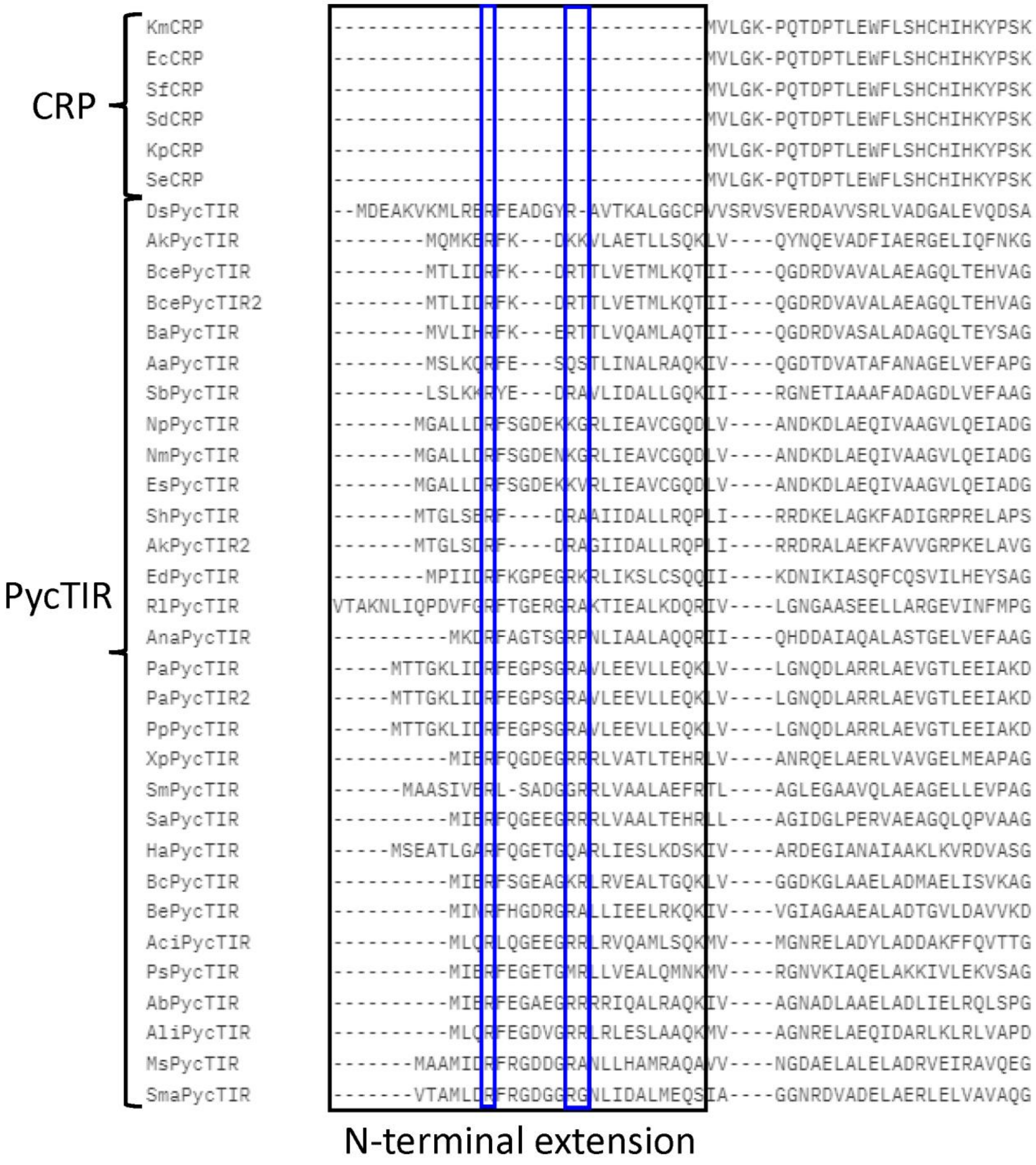

Supplementary Figure 9. Multiple sequence alignment of CRP family proteins and PycTIR proteins. The extra N-terminal sequences (21–30 a.a.) of PycTIR proteins are indicated by a black square. The conserved arginine residues at the N-terminal extension of PycTIR proteins are highlighted in blue.

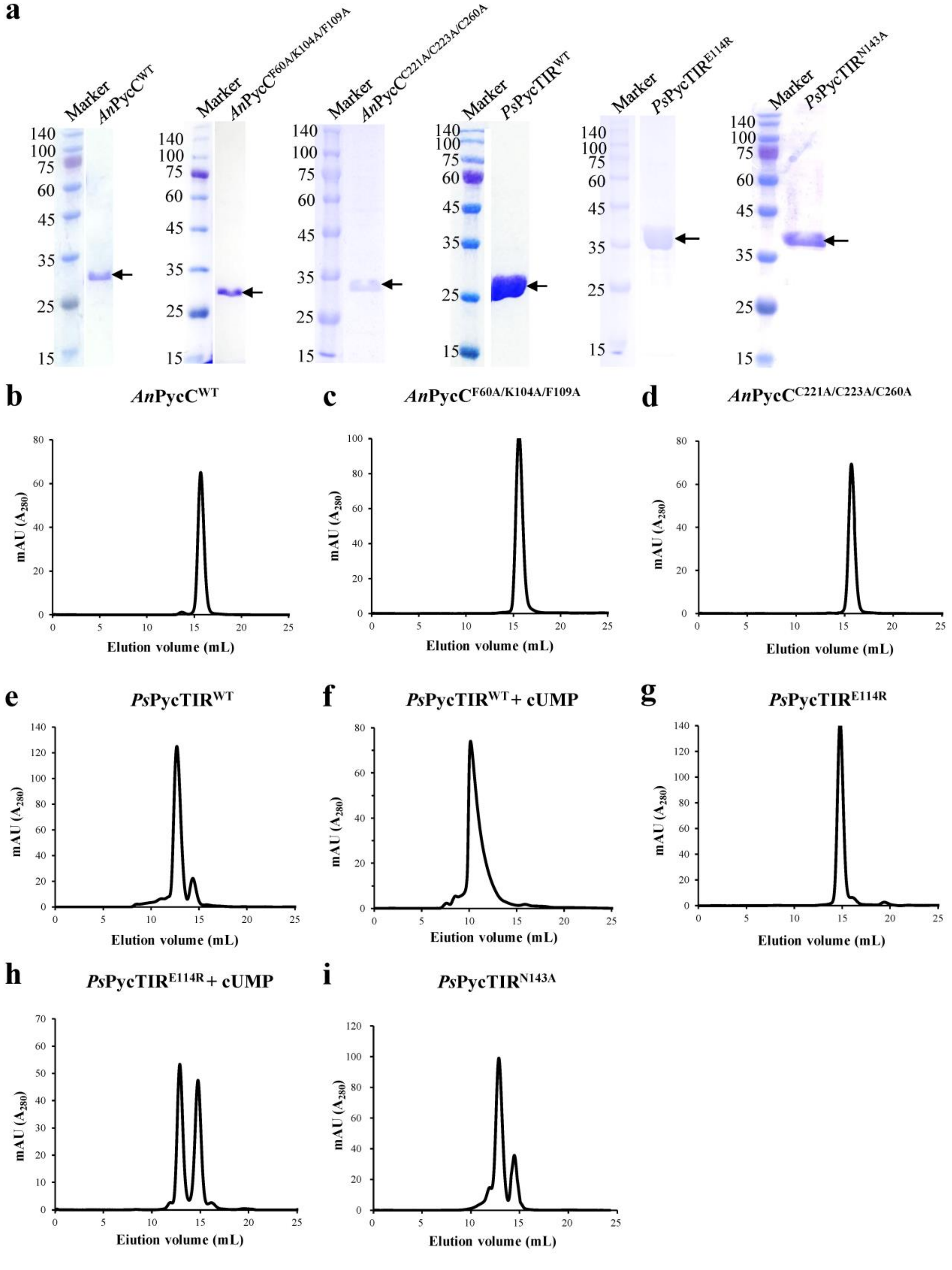

Supplementary Figure 10. SDS-PAGE and size exclusion chromatography profiles for all mutant proteins.

(a) 12 % SDS-PAGE analysis of purified *An*PycC^{WT}, *An*PycC$^{F60A/K104A/F109A}$, *An*PycC$^{C221A/C223A/C260A}$, *Ps*PycTIR^{WT}, *Ps*PycTIR^{E114R}, and *Ps*PycTIR^{N143A}. Each protein was purified to >95% purity. Source data are provided as a Source Data file. (b–i) Analytical size exclusion chromatography of (b) *An*PycC^{WT}, (c)

*An*PycC$^{F60A/K104A/F109A}$, (d) *An*PycC$^{C221A/C223A/C260A}$, (e) *Ps*PycTIR^{WT}, (f) *Ps*PycTIR^{WT} with cUMP, (g) *Ps*PycTIR^{E114R}, (h) *Ps*PycTIR^{E114R} with cUMP, and (i) *Ps*PycTIR^{N143A}. These SEC profiles indicate the proper folding and oligomerization of the purified proteins.

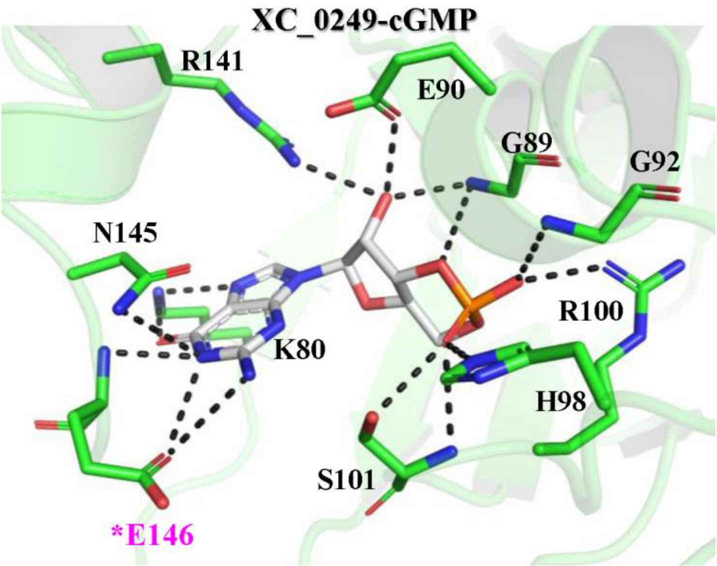

Supplementary Figure 11. Specific recognition of cGMP by CNBD-containing protein XC_0249 (PDB: 5H5O).

Enlarged view of the ligand-binding pocket of cGMP-bound XC_0249. The interacting residues are shown in sticks. The specificity-determining residue E146 is indicated with star and colored in magenta. The H-bonds are shown in black dashed lines.

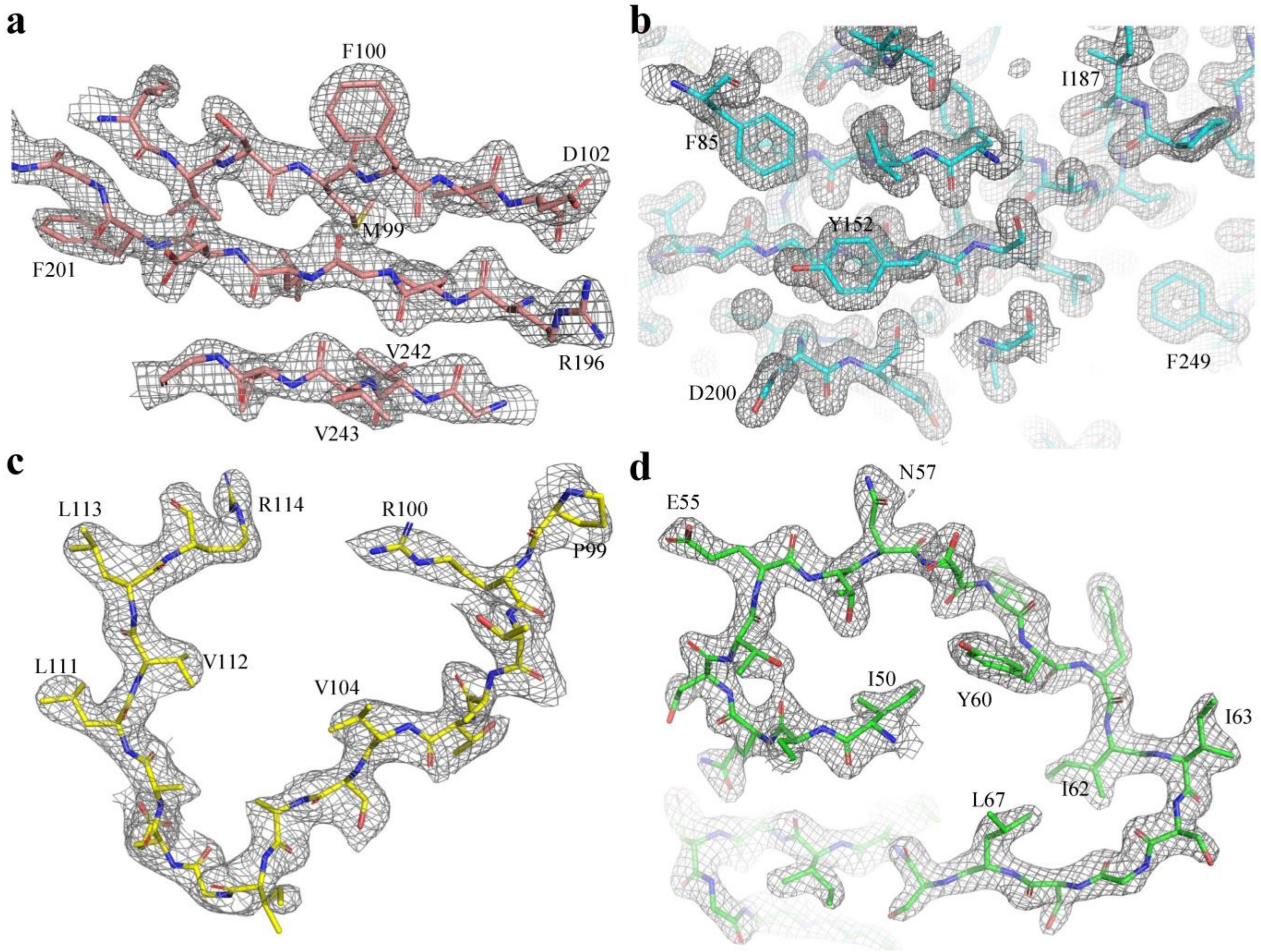

Supplementary Figure 12. Electron density maps superimposed on refined structures reported in this study.

Portion of the 2*F*o–*F*c electron-density maps of (a) *Ea*PycC, (b) *An*PycC, (c) *Np*PycTIR$_{CNBD}$, and (d) *Ps*PycTIR$_{CNBD}$–cUMP contoured at 1.5 σ. Selected residues of each crystal structure are indicated.
