# Supplementary material for: Structural and functional characterization of cyclic pyrimidine-regulated anti-phage system
Source: Nat Commun. 2024 Jul 4;15:5634. doi: 10.1038/s41467-024-49861-2 (PMC11224242; doi:10.1038/s41467-024-49861-2)
Supplement: Supplementary file 3 — Reporting Summary [file 41467_2024_49861_MOESM3_ESM.pdf]

nature portfolio

Corresponding author(s): Yeh Chen

Last updated by author(s): Jun 14, 2024

Reporting Summary

Nature Portfolio wishes to improve the reproducibility of the work that we publish. This form provides structure for consistency and transparency in reporting. For further information on Nature Portfolio policies, see our Editorial Policies and the Editorial Policy Checklist.

Statistics

For all statistical analyses, confirm that the following items are present in the figure legend, table legend, main text, or Methods section.

n/a	Confirmed	
☐	☒	The exact sample size (*n*) for each experimental group/condition, given as a discrete number and unit of measurement
☒	☐	A statement on whether measurements were taken from distinct samples or whether the same sample was measured repeatedly
☒	☐	The statistical test(s) used AND whether they are one- or two-sided<br>*Only common tests should be described solely by name; describe more complex techniques in the Methods section.*
☒	☐	A description of all covariates tested
☒	☐	A description of any assumptions or corrections, such as tests of normality and adjustment for multiple comparisons
☐	☒	A full description of the statistical parameters including central tendency (e.g. means) or other basic estimates (e.g. regression coefficient) AND variation (e.g. standard deviation) or associated estimates of uncertainty (e.g. confidence intervals)
☒	☐	For null hypothesis testing, the test statistic (e.g. *F*, *t*, *r*) with confidence intervals, effect sizes, degrees of freedom and *P* value noted<br>*Give P values as exact values whenever suitable.*
☒	☐	For Bayesian analysis, information on the choice of priors and Markov chain Monte Carlo settings
☒	☐	For hierarchical and complex designs, identification of the appropriate level for tests and full reporting of outcomes
☒	☐	Estimates of effect sizes (e.g. Cohen's *d*, Pearson's *r*), indicating how they were calculated

Our web collection on statistics for biologists contains articles on many of the points above.

Software and code

Policy information about availability of computer code

Data collection	HKL2000_v722
Data analysis	PHENIX 1.19.2-4158, Coot 0.9.6, PyMOL 2.3.3, UCSF ChimeraX 1.5, REFMAC 5.8.0267, NanoAnalyze v3.12.5, Bruker Compass Data Analysis software version 4.4

For manuscripts utilizing custom algorithms or software that are central to the research but not yet described in published literature, software must be made available to editors and reviewers. We strongly encourage code deposition in a community repository (e.g. GitHub). See the Nature Portfolio guidelines for submitting code & software for further information.

Data

Policy information about availability of data

All manuscripts must include a data availability statement. This statement should provide the following information, where applicable:

- Accession codes, unique identifiers, or web links for publicly available datasets
- A description of any restrictions on data availability
- For clinical datasets or third party data, please ensure that the statement adheres to our policy

Data are available within the article and supplementary information. The coordinates and structure factors of EaPycC, AnPycC, NpPycTIRCNBD, and PsPycTIRCNBD–cUMP complex generated in this study have been deposited in the Protein Data Bank under accession codes 8JSF [https://doi.org/10.2210/pdb8JSF/pdb], 8JSZ [https://doi.org/10.2210/pdb8JSZ/pdb], 8JSJ [https://doi.org/10.2210/pdb8JSJ/pdb], and 8JSK [https://doi.org/10.2210/pdb8JSK/pdb]. The protein structures used for analysis in this study are available in the Protein Data Bank under accession codes 1WC1 [https://doi.org/10.2210/pdb1WC1/pdb], 2CGP [https://

doi.org/10.2210/pdb2CGP/pdb], 2PTM [https://doi.org/10.2210/pdb2PTM/pdb], 6YII [https://doi.org/10.2210/pdb6YII/pdb], and 7R65 [https://doi.org/10.2210/pdb7R65/pdb]. The LC-MS dataset generated in this study has been deposited in the MassIVE repository under accession code MSV000094839 [https://doi.org/10.25345/C50R9MF94]. Protein sequences used in this study are available in the NCBI database under accession code WP_049037095.1 [https://www.ncbi.nlm.nih.gov/protein/WP_049037095.1] (EaPycC), WP_066377497.1 [https://www.ncbi.nlm.nih.gov/protein/WP_066377497.1] (AnPycC), WP_066377494.1 [https://www.ncbi.nlm.nih.gov/protein/WP_066377494.1] (AnPycTM), KZK76287.1 [https://www.ncbi.nlm.nih.gov/protein/KZK76287.1] (PsPycC), KZK76288.1 [https://www.ncbi.nlm.nih.gov/protein/KZK76288.1] (PsPycTIR), WP_081473994.1 [https://www.ncbi.nlm.nih.gov/protein/WP_081473994.1] (NpPycTIR), WP_080376336.1 [https://www.ncbi.nlm.nih.gov/protein/WP_080376336.1] (SmPycTIR), and EPR88376.1 [https://www.ncbi.nlm.nih.gov/protein/EPR88376.1] (AhPycTIR). Source data are provided as a Source Data file.

Research involving human participants, their data, or biological material

Policy information about studies with human participants or human data. See also policy information about sex, gender (identity/presentation), and sexual orientation and race, ethnicity and racism.

Reporting on sex and gender	N/A
Reporting on race, ethnicity, or other socially relevant groupings	N/A
Population characteristics	N/A
Recruitment	N/A
Ethics oversight	N/A

Note that full information on the approval of the study protocol must also be provided in the manuscript.

Field-specific reporting

Please select the one below that is the best fit for your research. If you are not sure, read the appropriate sections before making your selection.

☒ Life sciences ☐ Behavioural & social sciences ☐ Ecological, evolutionary & environmental sciences

For a reference copy of the document with all sections, see nature.com/documents/nr-reporting-summary-flat.pdf

Life sciences study design

All studies must disclose on these points even when the disclosure is negative.

Sample size	No statistical method was used to predetermine sample size. The sample sizes were chosen based on the previously reported protocols (Ko et al. Nature Communications. 2022, 13: 26; Hou et al. Nature Communications. 2023, 14: 8519).
Data exclusions	No data were excluded.
Replication	Replicates of all experiments were described in figure legends.
Randomization	Randomization was not applied to the functional experiments, because there was no sampling requirement. For refinements of X-ray crystal structures, the 5 % randomly selected R-free reflection set was determined by an internally built algorithm of PHENIX software.
Blinding	For biochemical experiments, blinding was not applicable because researchers need to verify samples and controls for each experiment. For crystallization experiments, blinding was not applicable because the aim of the experiment is to find more crystallization hits. For in vivo experiments, although the experiments were not formally blinded, they were assay as numerically coded constructs.

Reporting for specific materials, systems and methods

We require information from authors about some types of materials, experimental systems and methods used in many studies. Here, indicate whether each material, system or method listed is relevant to your study. If you are not sure if a list item applies to your research, read the appropriate section before selecting a response.

Materials & experimental systems

n/a	Involved in the study
☒	☐ Antibodies
☒	☐ Eukaryotic cell lines
☒	☐ Palaeontology and archaeology
☒	☐ Animals and other organisms
☒	☐ Clinical data
☒	☐ Dual use research of concern
☒	☐ Plants

Methods

n/a	Involved in the study
☒	☐ ChIP-seq
☒	☐ Flow cytometry
☒	☐ MRI-based neuroimaging
